# Challenges in Biomaterial-Based Drug Delivery Approach for the Treatment of Neurodegenerative Diseases: Opportunities for Extracellular Vesicles

**DOI:** 10.3390/ijms22010138

**Published:** 2020-12-25

**Authors:** Asit Kumar, Lina Zhou, Kaining Zhi, Babatunde Raji, Shelby Pernell, Erene Tadrous, Sunitha Kodidela, Anantha Nookala, Harry Kochat, Santosh Kumar

**Affiliations:** 1 Department of Pharmaceutical Sciences, University of Tennessee Health Science Center, Memphis, TN 38163, USA; lzhou13@uthsc.edu (L.Z.); pernellshelby@gmail.com (S.P.); etadrous@uthsc.edu (E.T.); skodidel@uthsc.edu (S.K.); 2 Plough Center for Sterile Drug Delivery Solutions, University of Tennessee Health Science Center, Memphis, TN 38104, USA; kzhi@uthsc.edu (K.Z.); braji@uthsc.edu (B.R.); hkochat@uthsc.edu (H.K.); 3 Covance Inc., Kinsman Blvd, Madison, WI 53704, USA; anfh3@mail.umkc.edu

**Keywords:** drug delivery, drug loading, biomaterial, extracellular vesicles, exosomes, microvesicles/microparticles, neurodegenerative disease

## Abstract

Biomaterials have been the subject of numerous studies to pursue potential therapeutic interventions for a wide variety of disorders and diseases. The physical and chemical properties of various materials have been explored to develop natural, synthetic, or semi-synthetic materials with distinct advantages for use as drug delivery systems for the central nervous system (CNS) and non-CNS diseases. In this review, an overview of popular biomaterials as drug delivery systems for neurogenerative diseases is provided, balancing the potential and challenges associated with the CNS drug delivery. As an effective drug delivery system, desired properties of biomaterials are discussed, addressing the persistent challenges such as targeted drug delivery, stimuli responsiveness, and controlled drug release in vivo. Finally, we discuss the prospects and limitations of incorporating extracellular vesicles (EVs) as a drug delivery system and their use for biocompatible, stable, and targeted delivery with limited immunogenicity, as well as their ability to be delivered via a non-invasive approach for the treatment of neurodegenerative diseases.

## 1. Introduction

Neurodegenerative diseases are characterized by malfunctions and the progressive death of neural cells with time. These diseases also exhibit complex and mixed clinical phenomena, including oxidative stress, neuroinflammation, and cell death [1]. The most commonly reported neurodegenerative diseases are Alzheimer’s disease (AD), Parkinson’s disease (PD), multiple sclerosis (MS), HIV-associated neurocognitive disorder (HAND), and traumatic brain injury (TBI) [2,3]. Current treatments for neurodegenerative diseases are not adequate to cure or defer disease development and progression [4]. The main reason for unmet medical needs is the presence of the complex and sophisticated blood–brain barrier (BBB). The human BBB is a highly selective, semipermeable brain barrier that offers a protective mechanism for the human brain. With the presence of multiple efflux transporters and tight junctions, certain drug molecules have difficulty crossing the BBB [5,6]. Therefore, it is critically important to design and develop biomaterial-based drug delivery systems to combat neuronal diseases.

## 2. Challenges and Implications of Biomaterials-Based Drug Delivery Approach in Neurodegenerative Diseases

Biomaterials have essential roles in the treatment of neurodegenerative diseases. Nanoparticles in particular have been used with higher frequency in research and development (Table 1) [7]. Among nanoparticles, different biomaterials were investigated in depth for their advantages and disadvantages [8]. Differentiated by monomer source, researchers worked on metal nanoparticles, metalloid nanoparticles, solid lipid nanoparticles (SLNs), polymeric nanoparticles, liposomes, and extracellular vesicles (EVs) [9]. There are many misunderstandings regarding nanoparticle nomenclature. “Nanoparticle” is a general name for drug delivery particles with a size between 10 and 1000 nm. Nanoparticles may be named after their shapes, e.g., nanocubes, nanoplates, nanorods, nanospheres, nanotetrapods, nanoprisms, and nanobelts [7]. Nanoparticles may also be named according to the specific materials used in the assembly, such as with solid lipid nanoparticles, Poly Lactic-co-Glycolic Acid (PLGA) nanoparticles, and metal nanoparticles. Hydrogels and macroscopic polymer matrix drug delivery systems have also been explored for the treatment of neurodegenerative diseases [10,11].

Metal nanoparticles are simple biomaterials with a straightforward structure. Gold nanoparticles (AuNPs) are mainly used as trackers rather than as drug delivery systems [13]. In stem cell therapies, AuNPs were used to visualize cell integration, cell proliferation, and tissue recovery [12]. Silver nanoparticles (AgNPs) have similar structures compared to AuNPs. However, AgNPs are used preferably as a therapeutic entity rather than as a drug delivery carrier. AgNPs can also trigger the anti-inflammatory reaction inside microglia, which can be beneficial in neurodegenerative diseases [14]. This mechanism may also be used to treat HAND, since microglia are a targeting site for antiretroviral drug delivery [5]. Metal oxide nanoparticles, such as iron oxide, cerium oxide, and zinc oxide, are developed for imaging and decreasing oxidative stress. These NPs respond to the magnetic field, which benefits researchers and health care providers [15]. Moreover, short hairpin RNA (shRNA) was reported to be immobilized onto iron oxide NPs in a PD model [16].

Quantum dots (QDs) have been widely used in the imaging and diagnosis of neurodegenerative diseases such as AD. QDs coated with amyloid-beta (Aβ) on the surface are used to track Aβ plaques [21]. Similar to AuNPs, QDs are also useful in tracking transplant cells and cell proliferation [22]. Metalloid nanoparticles are primarily made from cadmium-selenium QDs and silica. Silica nanoparticles loaded with or without drug were reported to incorporate into living neuronal cells [23] and the BBB in vivo [24,25].

SLNs and liposomes are made of similar materials with different structures [8]. SLNs use a single-layer spherical structure to encapsulate cargo molecules. In SLNs, lipids and cargos are uniformly mixed to form the core and then stabilized by surfactants. The final SLN usually has lipids and cargos in the core, with the surfactant’s hydrophilic segments facing outward to form the closure. SLNs can improve the stability and delivery of hydrophobic/lipophilic molecules [33]. In contrast with SLNs, liposomes utilize a bilayer spherical structure to encapsulate cargo molecules. Liposomes have a hydrophilic core surrounded by a hydrophobic/lipophilic layer, allowing liposomes to carry cargos with different physicochemical properties [27]. Several liposome-based drug products have been approved by the United States Food and Drug Administration (USFDA) [26]. In 2017, the USFDA approved a liposome-encapsulated combination of daunorubicin-cytarabine (Vyxeos) for adults with newly diagnosed therapy-related acute myeloid leukemia (t-AML) or AML with myelodysplasia-related changes (AML-MRC) (https://www.accessdata.fda.gov/drugsatfda_docs/label/2017/209401s000lbl.pdf). More importantly, the USFDA approved this product through the facilitated regulatory pathway, indicating the innovative nature of this drug delivery system [34,35]. Both SLNs and liposomes can be modified to provide additional features, such as site-specific targeting, lack of immunogenicity, and low toxicity. Micelles are another form of lipid nanoparticle. Micelles are very similar to liposomes and SLNs, but micelles have only one lipid layer without a lipid core. Micelle solution has been used to encapsulate curcumin for the potential treatment of several neurodegenerative diseases including AD, PD, and MS [28]. However, their application in humans is limited by their bioavailability, and therefore, the development of new nanoformulations with curcumin is necessary to treat neurodegenerative diseases. EVs, natural drug delivery systems, combine all the advantages from SLNs, liposomes, and micelles. EVs have been investigated in depth regarding targeted delivery, no/low toxicity, and personalized medicine [36]. The Covid-19 pandemic also brings EVs to researchers’ attention. Researchers are planning to take advantage of EVs to increase the dose of certain drugs while avoiding severe systemic toxicity [30].

Polymeric nanoparticles are the most advanced and well-developed drug delivery systems used in the treatment of central nervous system (CNS) diseases. Polymeric nanoparticles are made of polymers, which are macromolecules with monomers linked together to form a chain or chain-like structures [37]. Most monomers are naturally available in the human body. Hence, polymeric nanoparticles can be degraded and cleared out of the human body without triggering an immune reaction. The most popular polymeric nanoparticles are made of polylactic acid (PLA), PLGA, and polyethylene glycol (PEG) [7,9,37]. The porous structure of polymeric nanoparticles provides a large space to encapsulate cargos. PLGA nanoparticle-based drug formulations have been shown to cross the BBB and deliver cargos into both macrophages [6] and microglia [5]. Similarly, paclitaxel-loaded PLGA-NPs were also reported to cross the BBB with the assistance of glutathione coating [38]. Loperamide and rhodamine-123 coated NPs were also reported to be delivered into the brain [39].

Preferably, nanoparticle-based drug delivery systems for treating neurodegenerative diseases should be biocompatible, stable, and biodegradable. Production should be cost-effective, scalable, reproducible, and amenable to sterilization and functional modifications. Conversely, these nanoparticle systems should not be cytotoxic, neurotoxic, or provoke an immune response. Common inorganic materials used to form nanoparticle systems include iron, gold, silica, silver, titanium, and zinc [40]. Nanoparticles from these materials are amenable to different sizes and shapes and can be designed for diagnostic and therapeutic applications. These inorganic nanoparticle systems can cross the BBB. Their surfaces can be modified covalently or non-covalently to impart desired properties, such as targeted drug delivery and stimuli-responsive drug release. Inorganic nanoparticles can form reactive oxygen species and are less biodegradable [40]. With poor clearance, accumulation of the inorganic material or metabolites could lead to adverse effects. Inorganic nanoparticles fabricated from iron, silver, titanium, and zinc nanoparticle are less biocompatible due to their potential neurotoxicity [41,42,43]. Silica nanoparticles have been reported to reduce oxidative stress and dopamine levels in the striatum in rat models [44]. Animal model studies have shown that a decrease of the neurotransmitter dopamine in the striatum led to the loss of motor functions characterized by PD [45]. QDs are smaller nanoparticles typically made from a combination of metals and metalloids. QDs can also be made from organic materials such as graphene. QDs are mostly employed in bioanalytical applications, biological detection, and diagnosis, due to photoactivity and semi conductivity. The therapeutic applications of QDs are less explored. Some have reported the application of QDs as photosensitizers for photodynamic therapy (PDT) [46,47]. The generation of reactive oxygen species that are suspected of promoting neurodegeneration is typical during the PDT procedure, blocking the broad application of PDT [48]. To our knowledge, studies investigating the biodegradability of inorganic nanoparticles mainly focus on systemic clearance. Investigations into their distribution and long-term toxicity in the brain are essential for their deployment for therapeutic applications against neurodegenerative diseases.

Compared to inorganic nanoparticles, lipid and polymeric nanoparticles are inherently more biocompatible. In addition to the advantages over inorganic NPs, lipids and polymer nanoparticle surfaces can also be modified to impart desired functionalities. Natural polymer nanoparticles are generally less toxic and more biodegradable compared to synthetic polymers. The degradation of synthetic polymers can lead to increased toxicity. Without modifications, the hydrophobicity of some polymeric systems limits their application to the delivery of hydrophilic drugs. Hydrogels, on the other hand, are hydrophilic and thus are incompatible with hydrophobic drugs. If a controlled/long-term drug release is desired, polymeric nanoparticle systems are not recommended due to the burst release effect. The use of toxic organic solvents to produce polymer nanoparticle systems is sometimes unavoidable, thus limiting scalability. While most inorganic nanoparticle systems are used for diagnosis, several reports have demonstrated the use of lipid-based nanoparticle systems for therapeutic applications. The application of lipid nanoparticles toward the treatment of neurodegenerative diseases can be hindered by limited drug-loading capacity, especially with hydrophilic drugs and peptide/proteins, and reduced bioavailability due to faster clearance [49]. Lipid-based nanoparticle systems can also be susceptible to thermal, oxidative, or hydrolytic degradation. The surface modification capability of lipid-based nanoparticle systems is also limited due to the unavailability of diverse chemical functionalities and steric hindrance.

Naturally occurring EVs are a special kind of lipid-based nanoparticle that can be harvested from cell culture media, blood, plasma, and other bodily fluids such as cerebrospinal fluid (CSF), saliva, milk, and urine [50]. EVs can be considered biological products, and this categorization necessitates a special classification and regulatory scrutiny for approved clinical use. EVs consist of various biomolecules, including proteins and nucleic acids, which must be evaluated for cytotoxicity and immunogenicity. For EVs to be employed for therapeutic applications, their origin and biologic functions must be fully understood. The deployment of EVs for pharmaceuticals is also limited by challenges with the scalability of isolation techniques, inadequacies of current characterization techniques to address pharmaceutical and regulatory-relevant properties, and poor production reproducibility [51]. The application of EVs for therapeutics is in its infancy. Extensive investigations are needed to better understand their potential in drug development and unlock possible advantages over other drug delivery systems.

## 3. Extracellular Vesicle-Based Drug Delivery Systems

EVs are small membranous vesicles that are naturally produced and excreted from numerous cell types. EVs circulate through all bodily fluids and play a major role in intracellular and intercellular communication due to their multi-functional components such as proteins, DNAs, microRNAs, and mRNAs [52,53,54]. EVs’ ability to exchange genetic material with recipient cells can induce phenotypic modifications, making them a potential candidate in drug delivery systems for therapy [55]. Since EVs mediate cell-to-cell communication, they play critical roles in regulating the multiple facets involved in the pathogenesis of numerous diseases [56,57,58]. EVs may pathologically function as vehicles in drug treatment to disrupt communication pathways in pathogenesis to slow or alter disease progression [59].

### 3.1. EV Background

EVs are classified based on the size and biogenesis pathway. The three major classified subgroups of EVs are exosomes, microvesicles (MVs, also called microparticles), and apoptotic bodies, and their internal contents consist of lipids, proteins, and nucleic acids [60,61]. Exosomes, MVs, and apoptotic bodies all contain distinct proteins that indicate their biogenic pathways and specific functions [62]. Exosomes range from about 30–150 nm in diameter. However, exosome size distribution and zeta potential can vary significantly among preparations from different isolation methods [63]. Exosomes are formed by the inward budding of the membrane of early endosomes that eventually mature into multivesicular bodies [62]. Since exosomes originate in the endosomal pathway, they are enhanced with protein chaperones, scaffolding proteins, and proteins for endosomal trafficking [64]. Researchers initially believed that exosomes’ biological purpose was to expel unwanted material from cells. However, it has been discovered that they participate in cell maintenance, cell-to-cell communication, and tumor progression [59,62,65,66]. Microvesicles are derived from the outward budding of a cell’s plasma membrane [64,67], while apoptotic bodies are formed only during programmed cell death and generated through cell fragmentation and plasma membrane blebbing of apoptotic cells [67,68]. MVs can range from 50 to 1000 nm in size, and their formation is not well understood, but it is hypothesized that cytoskeleton components are required for their formation. Similar to exosomes, the biological purpose of MVs is to participate in cellular communication between local and distant cell types. Apoptotic bodies range from 50 to 5000 nm in size and are excreted by dying cells. They are formed when the cytoskeleton and plasma membrane separates due to increased hydrostatic pressure induced by cell contraction [62]. Various isolation methods have been developed to potentially overcome the challenges associated with EV isolation, such as EV heterogeneity and biochemical property overlap, that may inhibit effective EV isolation [69,70,71,72,73,74,75]. Some of the developed techniques include ultracentrifugation, density gradient centrifugation, exosome precipitation, antibody-based immunoaffinity purification, tangential flow filtration, and nano-flow cytometry. EV differentiation in various extracellular environments remains the main challenge of inefficient EV isolation in clinical settings and has to be overcome for these techniques to be clinically reproducible [62].

### 3.2. Drug Loading in EVs

Parental cells determine the biological structure and function of EVs in vivo. While some biological features of EVs are currently known, further exploration, especially about ensuring the safety and efficacy of drug-loaded EVs, must precede future therapeutic applications. The specific approaches used to load EVs will also affect drug-loading capacity and the lifetime of drug-loaded EVs Based on the understanding of the biological features of EVs, there are two major methods of drug loading: (I) endogenous drug loading and (II) exogenous drug loading. To minimize the elimination and degradation of EVs, the selection of drug loading method is pivotal.

#### 3.2.1. Endogenous Drug Loading

Endogenous drug loading methods aim to optimize drug cargo compartmentalization into EVs through the nonspecific binding of drugs to the cytoplasmic membrane of donor cells [76]. In this method, desired cargos are simply incubated with EV-secreting cells. Readily, cargos may passively diffuse across the cell membrane and these cells then secrete EVs loaded with the desired cargo [77,78]. This drug-loading technique is a relatively straightforward strategy that involves a step-by-step process to load drugs by manipulating the donor cells [76]. Endogenous drug-loading strategies rely on the natural mechanisms of EVs to package drug cargo more efficiently. For endogenous loading, donor cells are exposed to the drug of interest, which is followed by stimuli such as heat or hypoxia to induce the release of drug-loaded EVs [76]. EVs are hypothesized to be proficient candidates for drug delivery systems due to their endogenous origin and internal structures. EVs contain intrinsic biological functions and internal cage-like structures that are ideal for containing and delivering drug loads to specific molecular targets [79]. They also possess an aqueous core and lipophilic shell formed by the lipid bilayer, creating two internal compartments. The lipid bilayer gives EVs the amphiphilic nature that allows them to store and dissolve hydrophobic and hydrophilic compounds, making them desirable for use in drug delivery systems [80].

#### 3.2.2. Exogenous Drug Loading

To utilize EVs as drug carriers, an alternative approach for loading desired cargos into EVs can be achieved after EV isolation as an exogenous drug loading method. This method involves the isolation of EVs and subsequent drug loading or desired cargos in EV using mechanical approaches. Exogenous drug loading techniques vary depending on the target molecules of interest such as proteins, small molecules, and nucleic acids-specifically miRNA. Some of the mechanical methods used to load desired cargos into EVs include incubation at room temperature, electroporation, sonication, transfection, saponin permeabilization, and mechanical extrusion [78,81,82,83,84]. Electroporation, the guidance of proteins and signature sequences, producing hybrid EVs with lysosomes, transfecting donor cells, and transfection with commercialized reagents, are methods to load nucleic acids into EVs [81]. Drug-loading strategies include incubation, ultrasonic treatment, eddy current oscillation, and direct mixing [78,83,84]. Ultrasonic treatment was discovered to have increased the drug paclitaxel’s load capacity and supported the release of EVs excreted by macrophages [81]. The disadvantage of passive loading methods includes the degradation of exosomes due to multiple purification steps. The physicochemical properties of drug molecules can affect the stability and bioactivity of EVs [81].

While most cells produce EVs, not all cell-derived EVs are ideal drug carriers. Drug capacity and efficient delivery depend on the size, yield, intracavitary composition, and surface protein(s) [85]. EV biogenesis is a major factor determining the drug-loading capacity of the various types of cell-derived EVs. Since EVs encompass some of their parent cell contents during biogenesis, there is limited space for endogenous and exogenous drug loading [85]. Passive drug-loading strategies used in concentration gradient-based strategies, such as electroporation or sonication, result in low loading efficiency. To compensate for the low loading efficiency, researchers have opted for active loading strategies to target exosome membranes during exosome biogenesis [86]. Exosomes are enhanced with transmembrane proteins that can be fused to cargo molecules to localize these molecules in exosomal cytosol [86]. The heterogeneous internal components and chemical lipid composition can influence drug compatibility with EVs, affecting the drug-loading capacity. Pre-loading methods, post-loading methods, and drug hydrophobicity are factors influencing drug-loading capacity. For instance, researchers have found an 11-fold increase in drug-loading efficiency with the use of membrane permeabilizer saponin and hypotonic dialysis [85,87]. Researchers have taken a comparative approach to assess which method results in a higher loading efficiency. Porphyrins (of different hydrophobicities) served as the model drug that was encapsulated and loaded into EVs via dialysis, extrusion, electroporation method, and using saponin [87]. Hydrophobic compounds were loaded more efficiently in EVs via active methods than by using a passive incubation loading method [87]. Loading drugs into EVs resulted in a cellular uptake greater than 60%, and the photodynamic effect of hydrophobic porphyrins was greater in comparison to drug-loaded liposomes [87].

### 3.3. EV-Based Drug Delivery for Neurodegenerative Diseases

As we learn more about EVs and their potential roles in the human body, new opportunities for the use of EVs as therapeutic agents have risen. The ability of EVs to penetrate the BBB and transfer cellular components between the CNS and the peripheral circulatory system suggests promising applications in many neurodegenerative diseases, such as AD, PD, MS, HAND, TBI, as well as COVID-19-associated brain damage [53,88,89,90,91]. 

#### 3.3.1. AD

AD is a progressive neurodegenerative disease that is associated with dementia in the elderly [92]. AD pathogenesis is not yet completely clear. Many studies suggest that AD is characterized by the coexistence of two hallmark pathways that lead to the functional loss of synapses and neurons: the accumulation and disposition of insoluble Aβ plaques and the hyper-phosphorylation of tau proteins (P-tau), in addition to oxidative stress, cholinergic dysfunction, and inflammation [88,93,94]. Aβ plaque formation hinders synaptic plasticity, leading to neuronal apoptosis. This usually begins years before the appearance of any symptoms [92]. P-tau spread occurs after Aβ plaques are formed, and it is shown to affect specific sensors or motor functions in the brain, which is responsible for the loss of cognitive skills in AD patients [88,92]. Accumulating evidence suggests that EVs have a neurotoxic role in the propagation of AD, since amyloid precursor protein (APP)-metabolites, including Aβ, were found tied to exosomes, which are a subset of EVs [95].

Furthermore, Aβ plaques were found to be enriched with proteins that are associated with EV composition [95]. Studies have also indicated that the extent of neuronal loss is associated with EV levels in CSF [95]. EVs were also investigated in AD for their protective role as potential therapeutic agents [95,96]. For instance, cystatin C-loaded EVs have a neuroprotective role, and low serum cystatin C was detected in sporadic AD clinical presentation [95]. Different studies have suggested that EVs derived from human CSF may reverse the synaptic plasticity by disrupting the activity of Aβ plaques [95]. Another suggestion was to use short interfering RNA (siRNA)-loaded EVs against beta-secretase 1 to help decrease Aβ plaque formation [93,95]. Finally, studies have shown that the inhibition of EV release in AD animals using a neutral sphingomyelinase inhibitor (GW4869) has therapeutic benefits [97,98,99]. However, it also has undesired side effects associated with EV inhibition [97,100,101]. Nevertheless, the evidence is promising for EV use as early diagnostic biomarkers and potential therapeutic agents in AD.

#### 3.3.2. PD

PD is the second most common neurodegenerative disease among the elderly [102]. PD is a progressive movement disorder associated with neuronal death in different regions of the brain [103]. PD is characterized by the progressive loss of dopaminergic neurons in the substantia nigra pars compacta and over-expression and aggregation of misfiled α-Synuclein (α-Syn) proteins, which is the primary constituent of Lewy bodies [104,105]. α-Syn can be transmitted from either the CNS or peripheral blood monocytes to the brain via EVs. Even though PD is a neurodegenerative disease, α-Syn levels in blood erythrocytes were higher than in the CSF by about 10-fold [103]. The α-Syn proteins within peripheral red blood cells (RBCs) can cross the BBB and be taken up by microglia into the brain parenchyma via EVs (RBC-EVs) [103]. The same study has indicated that systemic inflammation increases BBB permeability, which in turn increases the RBC-EV influx, leading to the development of PD. Further, the uptake of RBC-EVs by microglia enhances microglial inflammatory responses, leading to an increase in neurodegeneration [103]. In a 6-hydroxydopamine (6-OHDA)-treated mouse model of PD, significant anti-inflammatory and neuroprotective effects were observed following the intranasal delivery of catalase-loaded EVs. The same approach can be explored to deliver therapeutic proteins across the BBB for the treatment of various neurodegenerative diseases [106].

To date, there is no curative treatment for PD, which is one reason why early diagnosis using EVs as biomarkers has been a major topic of interest in disorders such as PD [102,107]. The promising accumulation of evidence suggests that EVs can be loaded with therapeutic agents and engineered to target a specific neuronal population [102,108].

#### 3.3.3. MS

MS is a demyelinating autoimmune disease for which there is currently no effective re-myelination therapy [109]. However, a recent study found that in addition to EVs’ ability to cross the BBB, EVs are mediators in the axon myelination process [110]. In this study, EVs derived from mesenchymal stem cells (MSC-EVs) were demonstrated as feasible potential immunomodulatory agents and tissue repair mediators. Another study in 2019 has discussed the potential use of EVs as prognostic and diagnostic biomarkers for MS [111]. In the same study, an immune marker array was used to identify EV surface proteins to differentiate MS patients and healthy controls. Toll-like receptor-3 (TLR3) was found in lower concentrations in MS patients than in controls, while TLR4 was higher in MS patients [111]. Although it is still too early to say that EVs can be drug delivery agents or even diagnostic biomarkers in MS, the evidence is promising.

#### 3.3.4. HAND

HAND, which comprises different forms of neurocognitive impairments, is a growing concern among HIV populations [112]. Although the lifespan of people living with HIV has been prolonged since the discovery of antiretroviral therapy, HIV infection still promotes premature aging due to its persistent infection in the CNS glial cells, which can induce HIV-1 associated dementia [113,114,115]. EVs from HIV-1 infected cells were found to carry viral neurotoxins such as gp120, Nef, and Tat, which can cause cell death, BBB disturbance, as well as induce HAND [88,116,117,118,119,120]. EVs carrying HIV-1 Nef released from infected cells have been shown to promote latent HIV-1 reactivation [121,122]. Platelet and megakaryocyte-derived EVs render the cells susceptible to HIV-1 infection by transferring HIV co-receptors such as CXCR4 and CCR5 [123,124]. HAND presentation is very similar to AD, especially because AD hallmarks such as Aβ plaque accumulation and hyper-phosphorylated P-tau were detected in both AD and HAND [125]. However, in HAND, EVs carrying Tat may induce Aβ plaque accumulation, increase BBB permeability, inhibit the effect of Aβ peptide degrading enzyme (neprilysin), and inhibit the Aβ clearance mechanism by inhibiting the phagocytic activity of microglia [126,127,128].

EVs are being investigated to be used in HAND patients due to their ability to cross the BBB [129]. For instance, Tat-induced EV-Aβ and EV-tau could be drug targets for caffeine, as caffeine was studied to inhibit both Tat-induced Aβ production and tau phosphorylation [130,131]. Another example and potential intervention is the use of rapamycin-loaded EVs to modulate autophagy in the CNS in HAND patients. Early autophagy induction via rapamycin treatment showed promising results in reducing plaques, tangles, and cognitive deficits in preclinical AD models [132]. EVs are also explored as a potential diagnostic marker for cognitive impairment. Neuron-derived EVs are shown to be present at a significantly lower quantity in HIV individuals with neurocognitive impairment. Furthermore, these individuals had higher levels of high mobility group box protein 1 and Aβ in neuron-derived EVs [133].

#### 3.3.5. TBI

The use of EVs as diagnostic biomarkers also promises to fill the diagnostic accuracy gap in many different diseases, such as in TBI [134]. A recent study used miRNA EVs and GluR2^+^ EVs to identify TBI presence, severity, recovery, and history of prior injuries [135]. Moreover, AD biomarkers, such as Aβ and P-tau concentrations, were higher in EVs that were isolated from TBI patients than from controls [134,136]. Additionally, EV-tau and Aβ42 were higher in patients with a history of multiple TBIs. Furthermore, EV neurofilament neuropolypeptide and glial fibrillary acidic protein were associated with TBI diffusion and recovery [134]. Unresolved or dysregulated immune responses after TBI can contribute to chronic activation of neurotoxic microglia, eventually leading to progressive neuronal cell death [137]. Following TBI, activated microglia/macrophages release microparticles/microvesicles that propagate the injured brain’s neuroinflammatory responses by further activating the neighboring microglia [53]. Thus, inhibiting EV secretion is warranted to regulate the over-activated innate immune responses by microglia during brain injury. Studies indicate that glial cell activation and the regulation of innate immune responses can be achieved either by blocking EV biogenesis or by the neutralization of EVs using nSMase inhibitors and the novel surfactant polyethylene glycol telomere B, respectively [53,138].

#### 3.3.6. COVID-19 Associated Brain Damage

Mounting evidence suggests that the novel 2019 coronavirus disease (COVID-19) is neurotropic, as it is a viscerotropic disease [139]. COVID-19-related brain damage is associated with cytokine overproduction and toxicity, perhaps by delivering the virus and/or inflammatory/oxidative components to the CNS via EVs [30,139,140]. COVID-19 causes a broad variety of neurologic complications, such as hemorrhagic and/or ischemic strokes, seizures, and encephalopathy, which predicts a direct relationship between viral tropism and CNS injuries [139]. Presently, treatment options for COVID-19 are still being investigated, including the use of EVs as unique drug targets and carriers [30]. Moreover, EVs can be used in the treatment of COVID-19-associated brain damage due to their unique ability to penetrate the BBB and their potential to be engineered and targeted to a specific part of the CNS.

### 3.4. EV-Based Therapeutic Approach

EVs as therapeutic drug carriers are currently undergoing clinical trials for the treatment of pathogenic diseases such as cancer, autoimmune diseases, and neurodegenerative diseases [36,141]. EV-based drug delivery systems harness promising results due to their diverse cell-based origins and their ability to modulate various cell communication pathways (Table 2). Cargo loading methods, tissue targeting, the functional delivery of cargo to recipient cells, and the promotion of EV stability are strategic issues that are considered when choosing therapeutic agents for disease treatment [142]. Some therapeutic strategies may utilize EVs’ natural properties, such as pathogen suppression, immune modulation, or regeneration promotion, to improve the outcome of treatment by slowing pathogenesis or weakening autoimmune responses [142]. Data from recent clinical trials highlight that the importance of EVs as therapeutic delivery systems lie in the EV features, including cellular interactions, bio-distribution, circulation time, different cargo loading methods, and administration [143]. Drugs that could specifically benefit from EV drug delivery systems include anti-inflammatory agents and small RNA therapeutics [36,143,144,145,146,147,148,149].

Several human tumors originate in the epithelium and exhibit high epidermal growth factor receptor (EGFR) expression, hinting that EGFR could be a target in cancer drug delivery systems [150]. Nucleic acid drugs have promising therapeutic potential, but there are limits to their clinical application due to a lack of efficient drug delivery systems [151,152,153]. Ohno et al. demonstrated that exosomes can act as an effective drug delivery system of miRNA to EGFR-expressing breast cancer cells [150]. The profile and biocompatibility of exosomes make them ideal in miRNA drug delivery because they are the natural carriers of miRNA. In a preclinical study, MSC-EVs have been shown to promote neurogenesis, neurite remodeling, and synaptic plasticity in an experimental rat model of ischemic stroke [154], as well as improvement in sciatic nerve regeneration in rats [155]. Given the beneficial effects of MSC-EVs in many preclinical models, MSC-EV therapy was given to patients with graft-versus-host disease (GvHD). Shortly after the start of MSC-exosome therapy, clinical GvHD symptoms were found to be significantly improved [156]. Systemically administered MSC-EVs improved impaired function and structural injury in the fetal ovine brain following hypoxia-ischemia [157].

Monocyte-derived myeloid cells play a central role in inflammatory/inflammation-related autoimmune diseases. Studies have been conducted to test the efficiency of exosomes as drug delivery vehicles of anti-inflammatory agents [158]. Sun et al. examined EVs, specifically exosomes, as promising agents for delivering curcumin, an anti-inflammatory drug, to target inflammatory cells [158]. Curcumin is a natural polyphenol derived from the rhizome of Curcuma longa (turmeric) and known for its chemopreventative, antineoplastic, anti-inflammatory, and antioxidant activity [158,159]. However, curcumin’s low solubility remains a major issue due to its hydrophobic properties. To increase exosome encapsulation efficiency, curcumin was mixed with EL-4-derived exosomes, resulting in the solubility of exosomal curcumin five-fold higher than free curcumin [158]. In order to develop an exosomal-based delivery system to treat PD, a potent antioxidant enzyme catalase was loaded into exosomes ex vivo. Following intranasal administration, catalase-loaded exosomes demonstrated significant neuroprotective effects in an in vitro and in vivo model of PD [106].

## 4. Limitations of EV-Based Drug Delivery Systems and Current Advancements to Counter these Limitations

Exosomes have potential advantages in drug delivery. While the application of exosomes as drug delivery systems appears realistic in humans, there are still some challenges that lie ahead. For example, the problem of manufacturing large-scale batches of exosomes for clinical use remains unsolved. Furthermore, the production of homogenous EVs is challenging, as EVs produced from the same cell source have varied sizes [78]. These limitations need to be overcome in order to improve the feasibility of using EV-based drug delivery systems.

### 4.1. Interaction of Drugs with EV Components

Since EVs may serve as an ideal drug delivery system in CNS disease by crossing the BBB and delivering proteins, RNAs, DNA, and chemical drugs, various techniques are used for the loading of therapeutic agents inside EVs. However, various drug-metabolizing enzymes and drug transporters have been shown to be expressed in EVs [162]. These enzymes pose challenges in terms of the stability of drugs, especially small molecules, which can be metabolized and effluxed out of the EVs. Cytochrome P450 (CYP) enzymes are the major metabolizers of xenobiotics, including therapeutic drugs [162]. Recently, Kumar et al. have shown a significant expression of functional CYP enzymes in EVs derived from human plasma [163]. Further studies have demonstrated that various CYP enzymes, mostly expressed in the liver and immune cells, are packaged in EVs and eventually secreted in plasma [163]. Plasma EVs circulate in the periphery and perhaps in the CNS. They likely interact with other cell types by releasing these CYP enzymes. The further induction of CYP enzymes in cell-derived EVs, upon exposure to xenobiotics such as tobacco and alcohol, suggests the role of these EV CYP enzymes in drug metabolism in extrahepatic cells [164,165,166]. Similarly, efflux transporters, such as p-glycoprotein (P-gp) are expressed in cell-derived EVs and circulated via plasma [30]. These studies suggest that CYP enzymes and efflux transporters not only metabolize/transport xenobiotics in the liver and gut but can also clear toxic compounds, endogenous compounds, and therapeutic drugs in other extrahepatic cells/organs and the peripheral circulation. Therefore, a complete understanding of various EV components, including CYP enzymes and efflux transporters, is important to predict the stability of drugs encapsulated in EVs derived from various sources. Upon understanding the role of CYPs and transporters in EVs of various cell types, EVs can be isolated from a source that is devoid of these enzymes. Alternatively, drugs can be loaded along with CYP and P-gp inhibitors as pharmacoenhancers to inhibit/reduce the effects of these enzymes on the metabolic stability of loaded drugs. For example, being strong inhibitors of the CYP3A4 enzyme, ritonavir and cobicistat are known pharmacoenhancers of antiretroviral drugs [167], which can be used to co-formulate drugs that are CYP3A4 substrates.

In addition to efflux and metabolic stability, it is also important to study the interaction of small molecules and metals present in EVs with various drugs. For example, it is known that metals such as calcium interact with antibiotics such as doxycycline or minocycline, leading to reduced drug bioavailability [168]. Thus, antibiotics encapsulated in EVs isolated from milk (an easy source of EVs) are likely to interact with calcium and reduce the chemical stability of the drug. Similarly, EVs derived from lung alveolar macrophages may have various inhaled xenobiotics such as air pollutants, EVs derived from liver may have numerous stable xenobiotics obtained from food, and EVs derived from immune cells may have many small immune molecules. These small molecules are likely to interact with various drugs encapsulated in EVs isolated from their respective cells. Therefore, before developing EV-drug formulations, it is imperative to examine whether small molecules and metals present in EVs can interact with encapsulated drugs and reduce their stability. Thus, drug loading can be tailored to EVs isolated from cell types that do not have small molecules with the potential to interact with specific drugs.

Using multiple drugs simultaneously confers a high likelihood of producing drug–drug interactions (DDI) via CYP enzymes present in EVs. Many drugs are not only substrates, but they are also inhibitors and/or inducers/activators of CYP enzymes. Thus, DDIs occur as a result of the inhibition or induction/activation of CYP enzymes. Although most CYP enzymes are predominantly expressed in the liver, they are found to be packaged in EVs/exosomes and circulate with plasma. For example, CYP2E1, which is highly abundant in plasma EVs [168,169], causes a DDI between alcohol and acetaminophen, leading to hepatotoxicity. An overdose of alcohol and/or acetaminophen, as well as drinking alcohol while taking acetaminophen, will increase the risk of adverse drug reactions. Toxicity is not only expected in hepatocytes but also in extrahepatic cells, including CNS cells, via the circulation of EV CYP2E1. The expression of CYP enzymes also shows genetic variability. Thus, when using EVs/exosomes to deliver a drug, the interactions between drugs and the presence of polymorphic CYP enzymes should also be cautiously considered.

### 4.2. In Vivo Pharmacokinetics of EV Drugs

With greater attention paid to the potential of EV drug applications, a few EV drugs are currently undergoing clinical trials. The pharmacokinetics of EV drugs limit the efficacy and efficiency of EV drugs. Therefore, the administration method of EV drugs is an important step that governs the distribution of EVs into targeted sites or cells.

#### 4.2.1. Intravenous Injection (IV)

The size of EVs is one of the factors that determines the pharmacokinetics of EV drugs. When intravenous injection is performed, only EVs smaller than 100 nm diameter can move with RBCs, with a small fraction of accumulation near the vascular wall. This indicates that smaller EVs are more appropriate for drug delivery purposes. After intravenous injection into the mouse model, EV levels immediately reduce and aggregate in the liver [169]. As a result, the majority of EVs circulating with RBCs in vivo are cleared by the liver and the spleen.

DiD lipid dye-labeled MSC-EVs were injected into the tail vein of mice and were mainly captured by the liver, spleen, and bone marrow as observed in vivo imaging, with the strongest fluorescence in the liver and spleen of mice [170]. The biodistribution results from a different group show that the source of EVs is related to the distribution sites in mice [171]. The diameter of EVs has been shown to affect biodistribution, with EVs < 100 nm able to move through the liver without significant uptake by hepatocytes [172]. Blood concentrations and circulation kinetics of EVs also limit their pharmacokinetics. Macrophages identify the apoptotic signal on the EV membranes and promote clearance. Inhibiting the clearance of macrophages can maintain certain concentrations of EVs and prolong the circulation time of EVs in vivo [173].

#### 4.2.2. Intraperitoneal Injections, Subcutaneous Injections, and Oral Administration

Compared to intravenous injection, after the intraperitoneal or subcutaneous delivery of HEK293T exosomes, they accumulate in the liver, gastrointestinal tract, or pancreas [174]. It is feasible to increase the concentration and duration of exosome efficacy on the target organ by the local administration. The direct injection of EVs through intranasal delivery can prevent EVs from entering the systemic circulation and being cleared in large amounts by the liver and spleen [175]. Oral administration is one of the potential methods for EV administration without invasive injury. In contrast to intravenous injection, the oral administration of milk-derived exosomes improved immune function and reduced arthritis in mice [176].

### 4.3. Delivery of Drugs to the Target

The drug delivery system design depends on the structure of a drug molecule, its formulation, administration approach, dosage form, and other related techniques. The recent development in drug delivery systems focused on nanoparticles can increase the drug concentration in targeted parts of the body. Exosomes can be widely detected in many body fluids. Unlike other artificial nanoparticles, exosomes are naturally generated in vivo, which makes them an ideal drug delivery vehicle with a longer half-life, lower toxicity, and more specificity to targeted tissues [177,178]. Compared with synthetic drugs, drug-loaded exosomes are safer, stable, and biocompatible, due to their phospholipid bilayer structure [179]. The bio functional cargoes of exosomes range from nucleic acids, proteins, and lipids to synthetic drugs [180,181]. The uptake of exosomes has three steps: interactions between receptor and ligand, membrane fusion, and endocytosis/ phagocytosis [182]. The uptake of exosomes relies on one cell type and surface proteins of exosomes [183]. Some studies show that receptor–ligand interactions enhance the biological efficacy of exosomes, especially in cancer therapy [184] and modulating immune responses [182].

Researchers showed that exosomes delivered miRNA efficiently to EGFR-expressing breast tumor cells in a mouse study [150]. Due to this feature, exosomes can be potentially developed as diagnostic biomarkers. On the other hand, it has been proven that exosomes from neural stem cells recognized by the Ifbgr1 receptor on target cells are indispensable to maintaining the Signal transducer and activator of transcription 1 pathway’s activation [185].

### 4.4. Immune Clearance

Although the phospholipid bilayer structure of EVs makes them more biocompatible, elimination can happen before EVs arrive at the target cells. Most of the elimination is initiated by innate immunity. When exosomes are intravenously injected into mice, they are instantly cleared by the reticuloendothelial system before they reach the target tumor tissue [186]. Sinus CD169^+^ macrophages have been shown to suppress cancer progression by eliminating the tumor-derived exosomes before they interacted with B cells [187]. Exosomes can also regulate innate immunity. Tumor-derived exosomes can negatively regulate T cell immunity by raising adenosine levels when expressing CD39 and CD73 [188]. Thus, further study to understand the immune clearance of EVs, as well as the development of necessary steps to counteract immune clearance, is needed. One such approach could be the isolation of EVs from the plasma of patients, drug loading in these EVs, and injecting drug-loaded EVs back to the same patients. This personalized medicine approach may have the ability to eliminate/reduce unwanted and adverse immune reactions.

## 5. Conclusions

**Figure 1:**
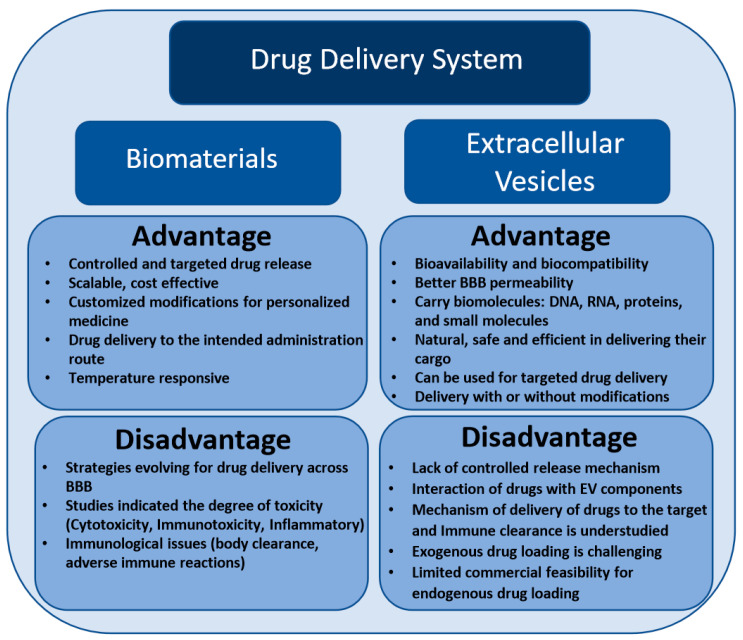
Possible advantages and disadvantages of biomaterials and extracellular vesicles as a drug delivery system.

This review presents recent advances in a biomaterial-based drug delivery approach for the controlled delivery of drugs or desired cargos to prevent or treat neurodegeneration in the CNS. As previously stated, biomaterials exist in different types; however, their use in clinical application is limited due to intensive interaction with the body tissue. Meanwhile, EVs possess numerous advantages over biomaterials in the context of a safe and effective drug delivery approach (Figure 1). In contrast to liposome-assisted drug delivery, EVs manifest higher loading efficiency and loading capacity for chemical drugs, and being natural nanoparticles, they are biodegradable and not expected to have adverse effects. The natural ability of exosomes to carry biological molecules such as long and short nucleic acid, proteins, and small molecules, as well as their ability to regulate gene expression and the phenotypic modifications of recipient cells make them an ideal drug-delivery modality. EVs not only demonstrate lower toxicity and lower immunogenicity than other drug delivery strategies but also bear specific surface proteins that can guide themselves to target organs. Due to the physiological role in various cellular functions, EVs have the potential to act as an ideal drug delivery system for neurodegenerative diseases by crossing the BBB and delivering desired cargos that include chemical drugs to the CNS. Under appropriate conditions, drugs that possess a hydrophobic or lipophilic nature or molecules such as antioxidants, anticancer, or anti-inflammatory drugs can be encapsulated into EVs. However, to intensify the bioavailability and efficacy of drugs with complicated properties, the successful integration of these drugs into EVs is required. In several studies, the delivery of target genes and selective silencing of genes aided by siRNA-loaded EVs has been validated. However, before we realize the full potential of EV-loaded drugs for therapeutic applications, certain limitations involving drug stability, in vivo pharmacokinetics, drug targeting, immune clearance, and the production of large-scale sterile preparations must be overcome.

## Figures and Tables

**Table 1:** Summary of popular biomaterials used in neurodegenerative diseases and related classification.

Material Classification	Name	Structure	References
Metal Elemental	Gold Nanoparticles (AuNPs)	Nanosphere	[12,13]
Metal Elemental	Silver Nanoparticles (AgNPs)	Nanosphere	[14]
Metal Oxide	Iron Oxide Nanoparticles	Nanosphere	[15,16]
Metal Oxide	Cerium Oxide Nanoparticles	Nanosphere	[17,18]
Metal Oxide	Zinc Oxide Nanoparticles	Nanosphere	[19,20]
Inorganic Compound	Quantum Dots	Nano Tube	[21,22]
Metalloid Elemental	Silica Nanoparticles (SiNPs)	Nanosphere	[23,24,25]
Lipid	Liposomes	Nanosphere with Bilayer Surface	[26,27]
Lipid	Micelles	Nanosphere	[28]
Lipid	Solid Lipid Nanoparticles (SLN)	Nanosphere with Monolayer Surface	[29]
Lipid	Extracellular Vesicles (EVs)	Nanosphere with Bilayer Surface	[30]
Polymer	Polylactic Acid (PLA)	Porous Nanosphere	[31]
Polymer	Poly Lactic-co-Glycolic Acid (PLGA)	Porous Nanosphere	[5,6]
Polymer	Polyethylene Glycol (PEG)	Porous Nanosphere	[32]
Polymer	Hydrogel	Porous Matrix	[10,11]

**Table 2:** Extracellular vesicles-based therapies in preclinical setting.

Loaded Drug Type	Targeted Disease Cells	Drug Delivery Method	Clinical Trial Phase	Reference
miRNA (nucleic acid drug type)	EGFR-expressing breast cancer cells	Exosome surface modification w/GE11 peptides	unspecified	[150]
Curcumin (anti-inflammatory)	Monocyte-derived myeloid cells associated with inflammation related autoimmune diseases	i.p. injection of exosomal curcumin into mice	unspecified	[158]
*Cas9* mRNA and antisense oligonucleotides	Leukemia cells and breast cancer cells	in vivo delivery of ASO-loaded EVs via i.p. injection in mice	unspecified	[160]
siRNA molecules	Cells affected by AD (neurons, microglia, oligodendrocytes)	Mice administered i.v. injection of RVG-targeted exosomes	unspecified	[108]
Catalase (antioxidant enzyme)	Cells of neurovascular unit affected by PD (neurons, astrocytes, and brain microvessel endothelial cells)	Intranasal administration of catalase-loaded exosomes	unspecified	[106]
siRNA molecules	α-Syn aggregates of Lewy bodies associated with Parkinson’s Disease	Peripheral injection of siRNA modified exosomes in the brain of mice	unspecified	[161]
